# Effect of Combined Equal-Channel Angular Pressing and Rolling on the Microstructure and Mechanical Properties of Zn-0.5Ag-0.2Mg Alloy

**DOI:** 10.3390/ma18122755

**Published:** 2025-06-12

**Authors:** Xiaoru Zhuo, Tiancheng Huang, Yuhan Xiong, Pengpeng Zuo, Xinyu Chen, Senlin Jin

**Affiliations:** 1 School of Materials Science and Engineering, Jiangsu University of Science and Technology, Zhenjiang 212100, China; xrzhuo@just.edu.cn (X.Z.); 19917929369@163.com (Y.X.); zuopp@just.edu.cn (P.Z.); cxy15618710325@gmail.com (X.C.); 2 Technology Innovation Center of Graphene Metrology and Standardization, State Administration for Market Regulation, National Institute of Metrology (NIM), Beijing 100029, China; huangtc1221@163.com; 3 National Industry Metrology and Measurement Center of Graphene Material (Shenzhen), Shenzhen Institute for Technology, NIM, Shenzhen 518107, China; 4 College of Materials Science and Engineering, Hohai University, Changzhou 213200, China

**Keywords:** Zn-Ag alloy, ECAP, rolling, mechanical property

## Abstract

Zn-Ag alloys are deemed extremely promising materials for manufacturing biodegradable medical implants. Nonetheless, their practical applications are still constrained by inferior mechanical properties. To tackle this issue, Zn-0.5Ag alloy was alloyed with Mg (0.2 wt.%) and processed by combined equal-channel angular pressing (ECAP) and rolling, with different rolling reductions (40%, 60%, and 75%). ECAP-processed Zn-0.5Ag-0.2Mg alloy exhibited superior mechanical properties to its as-cast counterpart. Subsequent rolling of 40% further enhances the mechanical performance of ECAP-processed Zn-0.5Ag-0.2Mg alloy, with yield strength (YS), ultimate tensile strength (UTS), and elongation (EL) reaching 255 MPa, 309 MPa, and 52%, respectively, surpassing the application requirements. As the rolling reduction increased to 60% and further to 75%, YS and UTS declined, whereas EL rose continuously. The underlying mechanisms for the variation in strength and ductility were elucidated based on microstructure evolution analysis through optical microscopy (OM), scanning electron microscopy (SEM), and electron backscatter diffraction (EBSD) characterizations.

## 1. Introduction

Biomedical implants play an indispensable role in treating various diseases [1,2]. Conventional implants are generally fabricated from corrosion-resistant metals such as Ti alloys [3] and stainless steel [4]. They commonly necessitate secondary removal surgery, boosting medical costs as well as the physical and mental suffering of patients [5,6]. Biodegradable medial implants have been proposed as a solution for addressing this issue. Zn alloys are promising candidate materials for fabricating biodegradable medical implants, owing to their merits such as moderate degradation rates and superior biocompatibility [7]. Recently, Zn-Ag alloys have emerged as a research hotspot due to their inherent antimicrobial capability, derived from the release of silver ions, thereby substantially mitigating infection risks [8,9,10].

Nonetheless, Zn-Ag alloys exhibit mechanical properties that are significantly inferior to the requirements of medical implants (YS ≥ 230 MPa, UTS ≥ 300 MPa, and EL ≥ 20%) [11]. Alloying and severe plastic deformation (SPD) are common strategies for enhancing the mechanical performance of metals. Zn-Ag alloys have been further alloyed with various elements including Cu [12,13,14,15,16], Zr [12,17], Mn [12,16,18,19], Mg [8,14,20,21,22,23,24,25], Ti [13], and Sc [26]. A variety of SPD techniques including ECAP [15,27,28,29,30,31], hot extrusion [8,12,17,18,32,33,34], and high-pressure torsion (HPT) [34,35] have been employed to process Zn-Ag based alloys. Alloying can lead to the formation of new phases such as (Ag, Cu)Zn_4_ [36], Zn_22_Zr [17], MnZn_13_ [18], Mg_2_Zn_11_ [8], and ScZn_12_ [26]. Fine precipitates strengthen Zn alloys through precipitation strengthening [37]. Second phase particles with a size larger than 1 μm promote dynamic recrystallization (DRX) during SPD through the particle-stimulated nucleation (PSN) mechanism [38]. SPD processing can induce significant grain refinement and dynamic precipitation of nanosized precipitates and increase dislocation density. Previous studies demonstrate that alloying and SPD enhance the mechanical performance of Zn-Ag alloys [5]. For example, hot-extruded Zn-0.1Ag-0.05Mg alloy exhibits a UTS of 247.8 MPa and an EL of 35% [8]. ECAP-processed Zn-2Ag alloy possesses an ultrahigh EL of 197% but a low UTS of 125 MPa [30]. HPT processed Zn-0.8Ag alloy shows a YS of 185 MPa [35]. Despite tremendous efforts, the strength and ductility of Zn-Ag based alloys developed so far rarely meet the application requirements.

SPD-processed Zn-Mg alloys commonly exhibit very high strength but poor ductility [5]. A hot-extruded Zn-1.6Mg alloy has a very high UTS of 365 MPa, but it is plagued by a low EL of 6% [39]. The low EL is related to a coarse Mg_2_Zn_11_ phase formed due to the addition of too much Mg. Inspired by this, a Zn-0.5Ag alloy was alloyed with minor Mg (0.2 wt.%) in this work. Previous studies [40,41] reported that combined ECAP and rolling endows hexagonal close-packed metallic materials such as Mg alloys with mechanical performance better than that of their counterparts processed by rolling or ECAP. Therefore, in this work, a Zn-0.5Ag-0.2Mg alloy was processed by combined ECAP and rolling, with an aim of enhancing its mechanical performance. Three different rolling reductions (40%, 60%, and 75%) were employed to investigate the influence of rolling reduction on the microstructure and mechanical properties of the Zn-0.5Ag-0.2Mg alloy. Interestingly, the Zn-0.5Ag-0.2Mg alloy processed by combined ECAP and rolling exhibits mechanical properties significantly superior to its counterpart processed by ECAP. The underlying mechanisms behind the variation in mechanical properties are discussed.

## 2. Experimental Procedure

In this work, to develop Zn-Ag-based alloys satisfying the application requirements of medical implants, Zn-0.5Ag alloy was further alloyed with 0.2 wt.% Mg and processed by combined ECAP and rolling, with different rolling reductions (40%, 60%, and 75%). The designed Zn-0.5Ag-0.2Mg alloy was prepared through conventional melting and casting techniques. The as-cast alloy was heat-treated and then processed by combined ECAP and rolling. Mechanical properties were evaluated by tensile tests. Microstructure characterization was performed by OM and SEM. The effect of rolling reduction on microstructure and mechanical properties was analyzed. The detailed experimental procedure is given as follows.

Raw materials for fabricating Zn-0.5Ag-0.2Mg alloy are pure Zn (99.99 wt.%), pure Ag (99.99 wt.%), and pure Mg (99.99 wt.%). They were dried at 200 °C for 12 h in a drying oven before use. Placed in a graphite crucible, raw materials were heated to 650 °C by a pit-type electric resistance furnace and held for 1 h at 650 °C. Subsequently, the melt was cast into a steel mold (with an inner dimension of 200 × 50 × 50 mm^3^). The graphite crucible and steel mold were coated with zinc oxide and then dried in a drying oven before use. The melting and casting processes were conducted under the protection of mixed CO_2_ (99 vol.%) and SF_6_ (1 vol.%). Subsequently, the obtained ingot was machined into 19.5 × 19.5 × 45 mm^3^ sized samples using wire-cut electrical discharge machining and then homogenized at 350 °C for 4 h. Homogenized samples were heated to 250 °C and underwent ECAP processing for 8 passes. ECAP was performed by rotary-die ECAP equipment [42]. ECAP-processed samples were named ECAP alloy. ECAP alloy was then rolled with three different rolling reductions (40%, 60%, and 75%) at room temperature. Samples rolled by 40%, 60%, and 75% were named ER 40%, ER 60%, and ER 75% alloy, respectively.

Tensile tests were performed by a universal testing machine (Suns UTM4294X, Jinan Hensgrand Instrument Limited Company, Jinan, China) with a strain rate of 10^−3^/s. Tensile test samples are dog-bone-shaped with a gauge length of 6 mm. An extensometer was used to measure the axial strain. Microstructure characterization was conducted by OM (Olympus BHM, Tokyo, Japan) and SEM (Hitachi Regulus8100, Tokyo, Japan) with an EBSD system and an energy-dispersive X-ray spectrometer (EDS). OM and SEM samples were prepared by mechanical grinding (using 180# to 2000# SiC abrasive papers), polishing (using 2.5 μm diamond paste), and etching (using a solution of H_2_O (10 mL), CrO_3_ (2 g), and Na_2_SO_4_ (0.15 g)). EBSD samples were electrolytic-polished at a current density of 450 mA/cm^2^ for approximately 4.5 min with a solution of C_2_H_5_OH (95%) and HClO_4_ (5%). EBSD data analysis was performed with HKL Channel 5 software. The grain sizes of as-cast and homogenized alloys were evaluated by the intercept method [43].

## 3. Results and Discussion

### 3.1. Microstructure Evolution

**Figure 1:**
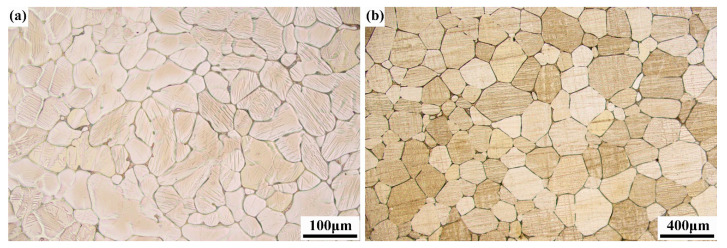
OM images of (**a**) as-cast Zn-0.5Ag-0.2Mg alloy and (**b**) homogenized Zn-0.5Ag-0.2Mg alloy.

**Figure 2:**
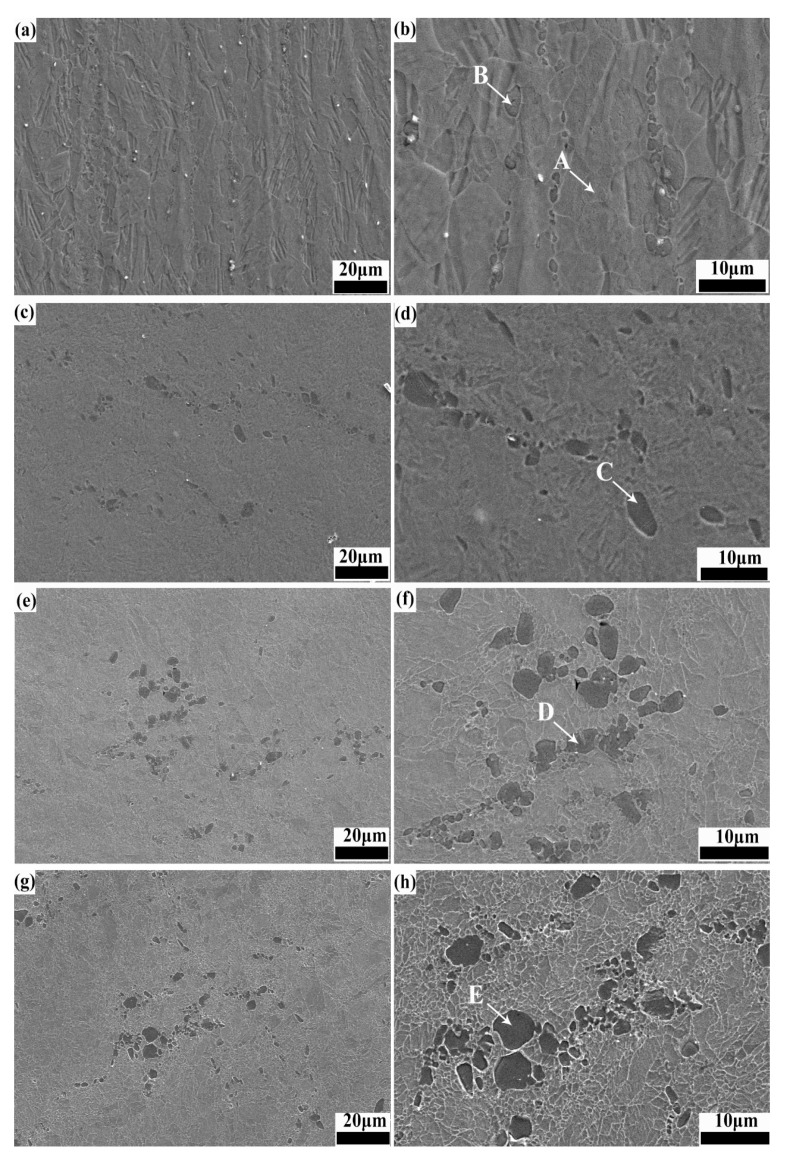
SEM images of (**a**,**b**) ECAP, (**c**,**d**) ER 40%, (**e**,**f**) ER 60%, and (**g**,**h**) ER 75% alloys.

OM images of as-cast and homogenized alloys are displayed in Figure 1. Grains of as-cast alloy are very coarse, possessing an average grain size (AGS) of approximately 51.8 μm. Homogenization induces grain growth, with the AGS increasing to 181.4 μm. SEM images of the ECAP, ER 40%, ER 60%, and ER 75% alloys are exhibited in Figure 2. The chemical composition of points A~E in Figure 2 is given in Table 1. Noticeably, numerous second-phase particles with a size ranging from submicron to micron exist in the ECAP, ER 40%, ER 60%, and ER 75% alloys, as marked by points B~E. The chemical composition of points B~E analyzed by EDS reveals that they correspond to a Mg_2_Zn_11_ phase. Owing to the negligible room-temperature solubility of Mg in Zn, minor addition of Mg to Zn and its alloys induces the formation of a Mg_2_Zn_11_ phase, as reported by previous studies [39,44].

**Figure 3:**
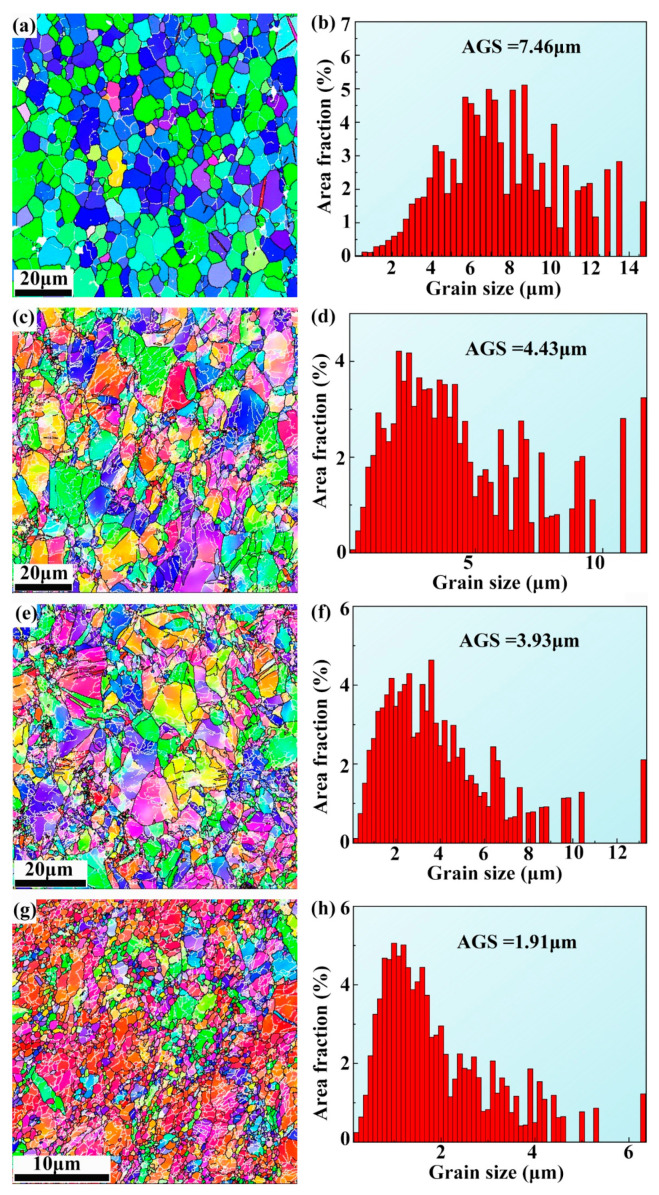
IPF of (**a**) ECAP, (**c**) ER 40%, (**e**) ER 60%, and (**g**) ER 75% alloys; grain size distribution histogram of (**b**) ECAP, (**d**) ER 40%, (**f**) ER 60%, and (**h**) ER 75% alloys.

EBSD images exhibiting inverse pole figures (IPFs) and grain size distribution histograms of ECAP, ER 40%, ER 60%, and ER 75% alloys are displayed in Figure 3. Note from Figure 3a that the microstructure of the ECAP alloy is dominated by equiaxed and fine grains, exhibiting an AGS of 7.46 μm, significantly smaller than that of the as-cast alloy. This significant grain refinement is attributed to DRX caused by ECAP. Plastic deformation-induced DRX of Zn alloys has been widely reported by previous studies [28,45]. Mg_2_Zn_11_ particles play a role in the DRX of the alloy. Micron-sized Mg_2_Zn_11_ particles promote DRX via PSN mechanism [38]. Moreover, submicron-sized Mg_2_Zn_11_ particles exert Zener pinning effect on grain growth, facilitating grain refinement [18]. Noticeably, subsequent rolling of 40% further refines grains, with the AGS reducing to 4.43 μm. In addition, as the rolling reduction increases to 60% and further to 75%, the AGS declines to 3.93 μm and further to 1.91 μm, respectively.

**Figure 4:**
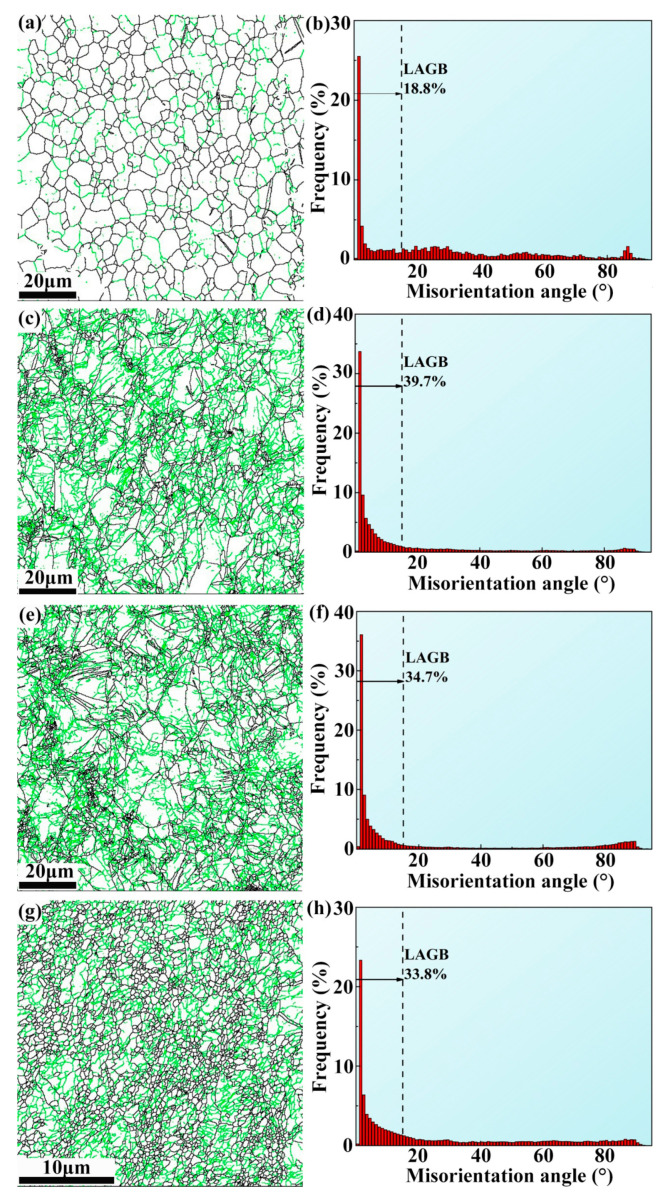
Grain boundary map of (**a**) ECAP, (**c**) ER 40%, (**e**) ER 60%, and (**g**) ER 75% alloys, where green lines represent low-angle grain boundaries (LAGBs, with a misorientation angle of 2°~15°) while black lines denote high-angle grain boundaries (HAGBs, with a misorientation angle exceeding 15°); misorientation angle distribution histogram of (**b**) ECAP, (**d**) ER 40%, (**f**) ER 60%, and (**h**) ER 75% alloys.

**Figure 5:**
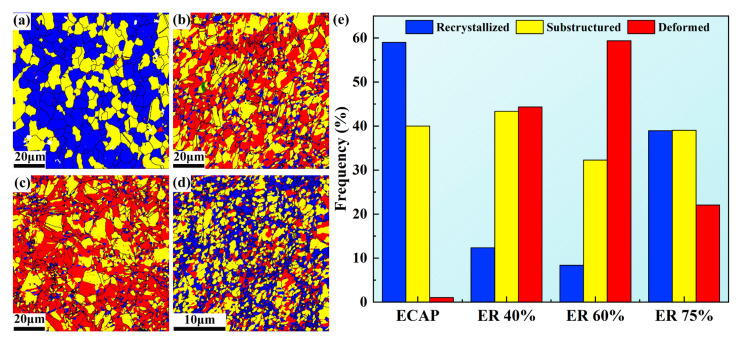
EBSD images displaying recrystallized (blue), substructured grains (yellow), and deformed grains (red) in (**a**) ECAP, (**b**) ER 40%, (**c**) ER 60%, and (**d**) ER 75% alloys; (**e**) histogram of different types of grains.

Figure 4 displays grain boundary maps and misorientation angle distribution histograms of ECAP, ER 40%, ER 60%, and ER 75% alloys. The low frequency (18.8%) of LAGBs in the ECAP alloy indicates a high degree of DRX, consistent with the equiaxed grains observed in the IPF image (Figure 3a). Indeed, the degree of DRX in the ECAP alloy is high, as displayed in Figure 5. The ER 40% alloy possesses the highest frequency (39.7%) of LAGBs. With the rolling reduction increases to 60% and further to 75%, the frequency of LAGBs decreases to 34.7% and 33.8%, respectively. The high frequency of LAGBs in the alloy processed by combined ECAP and rolling is ascribed to the high percentage of substructured and deformed grains formed by rolling.

**Figure 6:**
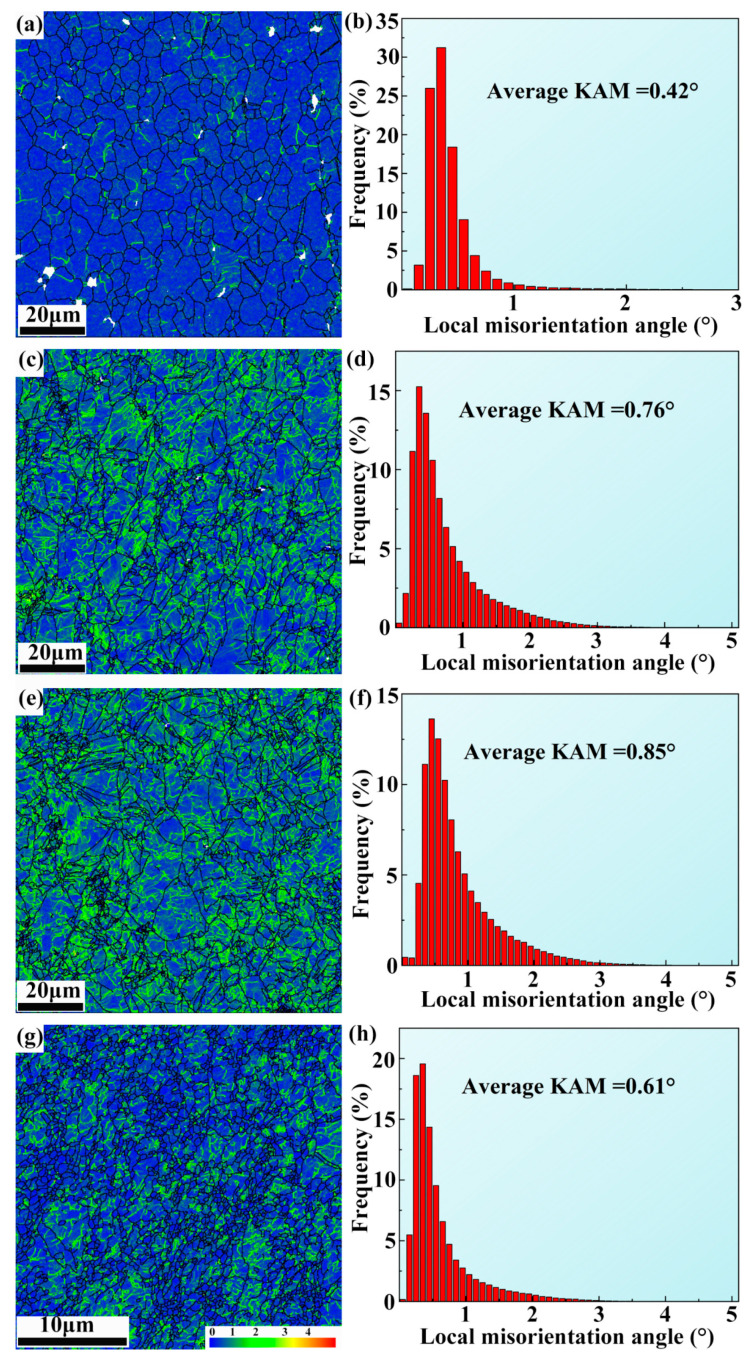
KAM map of (**a**) ECAP, (**c**) ER 40%, (**e**) ER 60%, and (**g**) ER 75% alloys; local misorientation angle distribution histogram of (**b**) ECAP, (**d**) ER 40%, (**f**) ER 60%, and (**h**) ER 75% alloys.

**Figure 7:**
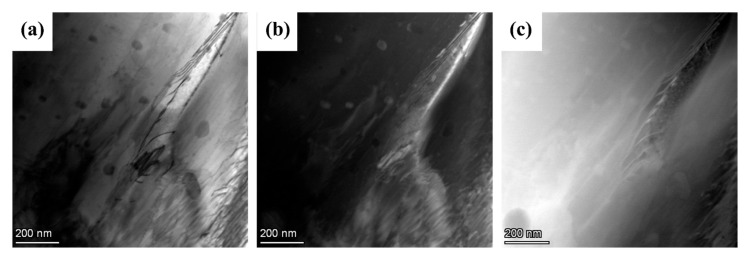
TEM images displaying dislocations in the ER 40% alloy (**a**) bright field, (**b**) dark field, and (**c**) high-angle annular dark field.

A kernel average misorientation (KAM) map and local misorientation angle distribution histogram of ECAP, ER 40%, ER 60%, and ER 75% alloys are exhibited in Figure 6. A KAM map is regarded as a measure of distribution of local lattice strain and dislocation, where elevated KAM values correlate with increased dislocation densities [46]. The ECAP alloy has a low average KAM of 0.42°, implying a low dislocation density. The low dislocation density is associated with the high degree of DRX of the ECAP alloy. DRX is a process involving the rearrangement and annihilation of dislocations [47]. Although numerous dislocations are generated during ECAP, they are mostly consumed by DRX. Therefore, the dislocation density in the ECAP alloy is low. Subsequent rolling of 40% significantly increases dislocation density, as implied by the greatly elevated average KAM of 0.76°. The variation in dislocation density is a result of the competition between dislocation formation by rolling and dislocation consumption by DRX. In cases of rolling reduction of 40% and 60%, dislocations formed by rolling overwhelm dislocations consumed by DRX due to the low degree of DRX. Consequently, dislocation density is higher than that in the ECAP alloy, as implied by increased average KAM values. TEM images exhibiting dislocations in the ER 40% alloy are displayed in Figure 7. As the rolling reduction increases to 75%, dislocation consumption by DRX overwhelms dislocation formation by increased rolling reduction, leading to decreased dislocation density.

### 3.2. Mechanical Properties

**Figure 8:**
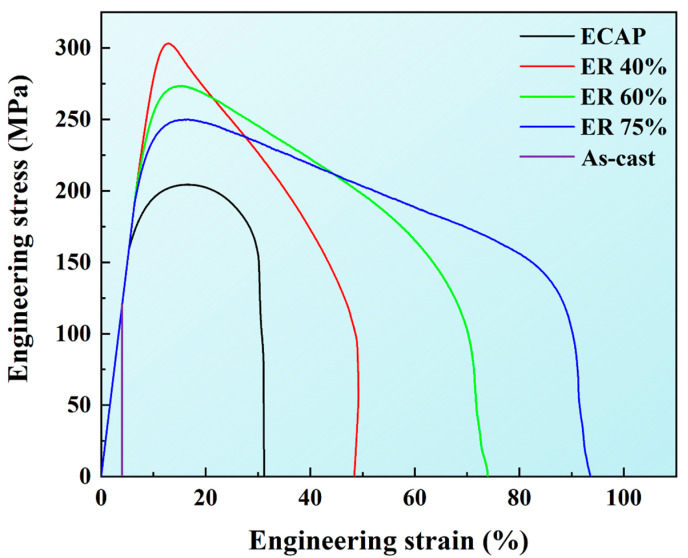
Engineering stress–strain curves of as-cast, ECAP, ER 40%, ER 60%, and ER 75% alloys.

Engineering stress–strain curves of as-cast, ECAP, ER 40%, ER 60%, and ER 75% alloys are displayed in Figure 8. The YS, UTS, and EL of them are shown in Table 2. The as-cast alloy exhibits quite poor mechanical properties, with a YS of 111 MPa, a UTS of 122 MPa, and an EL of 4%. ECAP significantly enhances the mechanical performance of the alloy, elevating the YS, UTS, and EL to 151 MPa, 197 MPa, and 32%, respectively. Subsequent rolling further improves mechanical properties. The ER 40% alloy displays a YS of 255 MPa, 309 MPa, and 52%, surpassing the requirements of medical implants. Interestingly, as the rolling reduction increases to 60% and further 75%, YS and UTS decline whereas EL rises continuously. The ER 40% alloy exhibits the best strength–ductility combination. A comparison on mechanical properties between ER 40% alloy and other recently developed Zn-Ag based alloys is given in Table 3. Noticeably, the mechanical properties of ER 40% alloy are excellent.

The main mechanisms for strengthening metals include fine grain strengthening, dislocation strengthening, solid solution strengthening, and precipitation strengthening [50]. The Hall–Petch equation is commonly employed to estimate fine grain strengthening. According to this equation, YS ($\sigma_{HP}$) is related to AGS (d) by

(1) $$\sigma_{HP}=\sigma_{0}+kd^{-0.5}$$

where $\sigma_{0}$ and k represent lattice friction stress and Hall–Petch slope, respectively [51]. The Bailey–Hirsch relation is generally used to evaluate dislocation strengthening $\sigma_{d}$. According to this relation, $\sigma_{d}$ is associated with dislocation density (ρ) by

(2) $$\sigma_{d}=M\alpha Gb\sqrt{\rho }$$

where M, α, G, and b denote the Taylor factor, constant, shear modulus, and Burgers vector of basal slip, respectively [52]. It is obvious from Equation (2) that a higher dislocation density results in stronger dislocation strengthening. Solid solution strengthening ($\sigma_{s}$) correlates with lattice distortion caused by solute atoms, which induces increased dislocation motion resistance [53]. It is evaluated by

(3) $$\sigma_{s}=k_{s}^{\frac{1}{n}}c^{n}$$

where $k_{s}$ is the strengthening coefficient, n denotes a positive exponent, and c represents solute atom concentration [35]. Note from Equation (3) that solid solution strengthening is positively correlated with solute atom concentration. Precipitation strengthening ($\sigma_{p}$) is widely attributed to the Orowan mechanism, wherein dislocations bypass non-shearable precipitates. It is estimated by

(4) $$\sigma_{p}=\frac{0.13Gb}{\lambda }ln\frac{d_{p}}{2b}$$

where λ and $d_{p}$ denote the average distance between precipitates and the average size of precipitates, respectively [37].

The ECAP alloy possesses significantly refined grains compared to the as-cast alloy. The tremendously enhanced YS of the ECAP alloy is mainly ascribed to fine grain strengthening. Compared to the ECAP alloy, the ER 40% alloy has finer grains (Figure 3) and higher dislocation density (Figure 6). Consequently, the higher YS of the ER 40% alloy mainly derives from fine grain strengthening and dislocation strengthening. As the rolling reduction increases to 60%, grains are further refined (Figure 3) and, meanwhile, the dislocation density is further elevated (Figure 6). Based on Equations (1) and (2), the ER 60% alloy should have a higher YS than the ER 40% alloy. Nonetheless, unexpectedly, the YS of the ER 60% alloy is lower. Previous studies ascribed such a grain refinement softening phenomenon to grain boundary sliding (GBS) [35]. With the further increase in rolling reduction to 75%, grains are finer, and dislocation density is lower. Therefore, the decreased YS of the ER 75% alloy is attributed to grain refinement softening and decreased dislocation strengthening. In contrast to YS, EL exhibits a rising trend with increasing rolling reduction, which is related to enhanced GBS activity induced by decreasing grain size. GBS can tremendously improve ductility and even lead to superplasticity [28]. In addition, the decreasing dislocation density with increasing rolling reduction also contributes to increasing EL.

## 4. Conclusions

In this study, a Zn-0.5Ag-0.2Mg alloy was processed by combined ECAP and rolling with three different rolling reductions (40%, 60%, and 75%). ECAP significantly refines grains and thus enhances the mechanical properties of the as-cast alloy. Subsequent rolling further refines grains and improves mechanical performance. The AGS exhibits a decreasing trend with increasing rolling reduction. Interestingly, YS and UTS decrease while EL increases as the rolling reduction increases from 40% to 60% and further to 75%. ER 40% exhibits the best mechanical performance with a YS of 255 MPa, 309 MPa, and 52%, surpassing the requirements of medical implants. Strength enhancement is mainly ascribed to grain refinement strengthening and dislocation strengthening. The decrease in strength with increasing rolling reduction derives from the grain refinement softening induced by grain boundary sliding. This study offers valuable insights into the development of high-performance Zn alloys for biomedical and industrial applications. Nonetheless, the corrosion rate, which is an important factor to be considered when designing biodegradable medical implants, was not investigated in this work. Future attention should be paid to the corrosion behavior of the developed Zn-Ag-Mg alloys.

## Figures and Tables

**Table 1:** Chemical composition of points A~E in Figure 2 analyzed by EDS (in atomic percentage).

Point	Zn	Ag	Mg	Phase
A	98.98	0.58	0.44	η-Zn
B	80.40	0.59	19.01	Mg_2_Zn_11_
C	77.15	0.96	21.89	Mg_2_Zn_11_
D	79.10	0.79	20.11	Mg_2_Zn_11_
E	81.02	0.51	18.47	Mg_2_Zn_11_

**Table 2:** YS, UTS, and EL of as-cast, ECAP, ER 40%, ER 60%, and ER 75% alloys.

Alloy State	YS (MPa)	UTS (MPa)	EL (%)
As-cast	111 ± 4	122 ± 5	4 ± 3
ECAP	151 ± 12	197 ± 14	32 ± 5
ER 40%	255 ± 4	309 ± 8	52 ± 6
ER 60%	222 ± 7	269 ± 6	71 ± 12
ER 75%	212 ± 3	257 ± 5	80 ± 14

**Table 3:** A comparison on mechanical properties between ER 40% alloy and other recently developed Zn-Ag based alloys.

Composition	Fabrication Method	YS (MPa)	UTS (MPa)	EL (%)	Ref.
Zn-0.5Ag-0.2Mg	ER 40%	255	309	52	This work
Zn-0.8Ag	HE	114	160	18	[27]
Zn-0.8Ag	ECAP	76	96	143	[27]
Zn-1Ag	HE	136	183	28	[17]
Zn-2Ag	HE	192	237	37	[48]
Zn-2Ag	ECAP	100	125	197	[30]
Zn-2.5Ag	HE	147	203	35	[33]
Zn-4Ag	HE		228	27	[18]
Zn-4Ag	HR	182	222	52	[26]
Zn-4Ag	CR	123	141	157	[16]
Zn-5Ag	HE	210	252	37	[33]
Zn-7Ag	HE	236	287	32	[33]
Zn-0.05Ag-0.05Mg	HE	164	180	9	[8]
Zn-0.1Ag-0.05Mg	HE	204	245	35	[8]
Zn-1Ag-0.05Zr	HE	166	211	35	[17]
Zn-1.5Ag-1.5Cu	HE	164	222	36	[36]
Zn-2Ag-1.8Au-0.2V	HR	168	233	17	[49]
Zn-4Ag-0.1Sc	HR	202	261	73	[26]
Zn-4Ag-0.2Mn	HE		267	25	[18]
Zn-4Ag-0.4Mn	HE		281	29	[18]
Zn-4Ag-0.6Mn	HE		302	36	[18]
Zn-4Ag-1Cu	CR	150	169	133	[16]
Zn-4Ag-1Mn	CR	162	207	91	[16]

HR: hot rolling; CR: cold rolling.

## Data Availability

The data that support the findings of this study are available from the corresponding author on reasonable request.
